# Height, body mass index, and prostate cancer: a follow-up of 950 000 Norwegian men

**DOI:** 10.1038/sj.bjc.6601206

**Published:** 2003-09-30

**Authors:** A Engeland, S Tretli, T Bjørge

**Affiliations:** 1 Division of Epidemiology, Norwegian Institute of Public Health, PO Box 4404 Nydalen, N-0403 Oslo, Norway; 2 The Cancer Registry of Norway, Institute of population-based cancer research, N-0310 Oslo, Norway; 3 Department of Pathology, The Norwegian Radium Hospital, N-0310 Oslo, Norway

**Keywords:** prostate cancer, cohort study, Norway

## Abstract

The present study explored body mass index (BMI), height, and risk of prostate cancer in a large Norwegian cohort of 950 000 men aged 20–74 years, whose height and weight were measured in a standardised way in the period 1963–1999. These were followed for an average of 21 years. The Cox proportional hazard models were used in the analyses. During follow-up, 33 300 histologically verified cases of prostate cancer were registered. The risk of prostate cancer increased by both BMI and height. The magnitude of the increase by BMI was modest, the relative risk (RR) of obese men (BMI⩾30) compared with normal weighted was 1.09 (95% CI: 1.04–1.15). However, the RR at age 50–59 years was 1.58 (95% CI: 1.29–1.94) in men being obese at about age 45 years compared with normal weighted men. The tallest men had an RR of 1.72 (95% CI: 1.46–2.04) compared with the shortest men. The overall effect of BMI on the incidence of prostate cancer was modest. The larger effect found in men aged 50–59 years might partly explain the previous inconsistent findings.

Worldwide, prostate cancer is the third most common malignancy in men with an estimated number of 500 000 cases in the year 2000, three-quarters in men aged 65 years or older (Parkin *et al*, 2001). Around 1990, the incidence of prostate cancer increased in many countries due to the introduction of widespread blood testing with prostate-specific antigen (PSA) (Parkin *et al*, 2001). In Norway, testing with PSA was introduced at the beginning of the 1990s (Harvei, 1999).

The aetiology of prostate cancer is largely unknown (Hsing *et al*, 2001). However, hormonal relations, age, and race are known risk factors. The relation between age and the incidence of prostate cancer is especially strong. The large age-gradient, combined with the ageing of many Western populations, will make prostate cancer an increasing health problem.

Certain studies on body mass index (BMI) and prostate cancer risk have concluded that there is no association (Bianchini *et al*, 2002). In a review of the literature up to 1998 (Bergström *et al*, 2001), however, it was concluded that elevated BMI was associated with the risk of prostate cancer. Overweight men (25<BMI<30; BMI=(weight in kilograms)/(height in metres)^2^) had a 6% increased risk compared with normal-weighted men (BMI 20–25), while obese men (BMI⩾30) had a 12% increased risk. The proportion of prostate cancer attributable to overweight and obesity in European men was estimated to be 4% (Bergström *et al*, 2001). A review of 12 case–control studies and 11 cohort studies (Nomura, 2001), revealed the inconsistencies between studies on this issue. If the relative risks (RRs) found by Bergström *et al* are close to the ‘real’ level, the increased risk of prostate cancer associated with overweight/obesity is small. Large and well-conducted studies are necessary to reveal increases in the risk of this magnitude.

Also, the findings in studies on the association between height and prostate cancer is inconsistent; two of nine cohort studies found a significant positive association between height and prostate cancer, but none of six case–control studies (Nomura, 2001).

The aim of the present study was to explore the relation between height and BMI and the risk of prostate cancer in a large Norwegian cohort with height and weight measurements in the age 20–74 years.

## MATERIAL AND METHODS

### Study population

During 1963–1975, height and weight were measured as part of a screening programme aimed at detecting tuberculosis in the general Norwegian population (Waaler, 1984; Bjartveit, 1997). This mass examination was compulsory for persons aged 15 years and older, and enrolled 1.7 million people. The attendance was about 85% in persons above the age of 15 years (Waaler, 1984). Previous reports have described the impact of adults’ height and weight on mortality (Waaler, 1984), and the material has also been utilised to explore relations between BMI and cancer incidence (Helseth and Tretli, 1989; Tretli, 1989; Tretli and Magnus, 1990; Thune *et al*, 1993; Robsahm and Tretli, 1999; Tretli and Robsahm, 1999). In 1963–1964 and in 1972–1999, height and weight were also measured in health surveys in different parts of Norway (Bjartveit *et al*, 1979; Bjartveit, 1997). The attendance in the mid-1970s was 85–90%, but decreased to about 75% in the mid-1990s (Bjartveit, 1997). The complete cohort has recently been used in a study of the impact of height and BMI on mortality (Engeland *et al*, 2003).

The body weight (kilograms) was measured on scales that were calibrated regularly, and registered to the nearest half kilogram. Body height was measured and noted to the nearest centimetre. The height was measured without shoes, and weight was measured with the subject wearing light clothing. If the height and weight measurements were performed under irregular circumstances, the measurement was excluded (the persons were wearing shoes, the persons were disabled, etc.). Further, measurements without registered height or height below 120 cm, or without registered weight or weight below 20 kg were excluded (67 measurements).

In the present study, the earliest accepted measurement between age 20 and 75 years was used for each man. There were 952 142 men with such measurements.

The study cohort was linked to the Death Registry at Statistics Norway to follow all persons in the present study from the date of measurement until eventual emigration or death. A unique 11-digit identification number assigned to all individuals living in Norway after 1960 simplified the linkage.

All cases of prostate cancer (International Classification of Diseases, seventh revision (ICD-7): 177) in the study cohort were identified by linkage to the Cancer Registry of Norway. Cancer registration, covering the entire Norwegian population of about 4.5 million inhabitants (2002), was initiated in 1952, and reporting of all diagnosed cancer cases has since been compulsory by law. Notifications are independently issued by all clinical and pathology departments involved in the diagnosis and treatment of cancer. In addition, all death certificates with a mention of premalignant or malignant disease reach the registry. The quality of the prostate cancer data in the Cancer Registry of Norway has been assessed, finding an error rate of about 1% of all data elements (Harvei *et al*, 1996).

In the present study, only histologically verified prostate cancer diagnoses were included. Men with a prostate cancer diagnosis prior to the height and weight measurements were excluded (676 men). The men in the cohort were followed up from the date of measurement until date of prostate cancer diagnosis, emigration, age 100 years, death, or until 30 June 2001. Altogether, 951 466 men were eligible for the study. A small number of these (seven men) were lost to follow-up.

### Statistical methods

The Cox proportional hazards regression models (Cox and Oakes, 1984), with time since measurement as time variable, were fitted to obtain RR estimates of prostate cancer for different groups. In the analyses, the following categorised variables were included:

1. age at measurement: 20–24 years,…, 65–69 years, and 70–74 years.
2. year of birth: <1900, 1900–1909,…, 1940–1949, and ⩾1950.
3. BMI ((weight in kilograms)/(height in metres)^2^):
  - detailed categorisation: BMI <18.50, 18.50–19.49,…, 22.50–23.49, 23.50–24.99, 25.00–27.49,…, 32.50–34.99, and ⩾35.00.
  - WHO categorisation (World Health Organization Consultation on Obesity, 1998): BMI <18.50 (underweight), 18.50–24.99 (normal range), 25.00–29.99 (preobese/overweight), and ⩾30.00 (obese).
4. height (cm): <160, 160–169, 170–179, 180–189, and ⩾190.

Tests for linear trend were calculated by including BMI and height, respectively, as continuous variables.

The proportionality assumption in the Cox model was assessed by inspecting log-minus-log plots, results from stratified analyses, and results from separate analyses for different intervals of observation time. To explore whether BMI had a different impact on prostate cancer in the different age groups, analyses stratified on attained age and age at measurement were performed.

All analyses were carried out with the statistical program package SPSS (SPSS Inc., 2001). The results were presented as RRs of prostate cancer with 95% confidence intervals (CI).

## RESULTS

A total of 951 459 men (mean age: 44. 5 years) were followed for an average of 21.0 years (range 0–38 years), comprising 20 010 705 person-years (Table 1

**Table 1:** Number of observed prostate cancer cases, person-years, and overall prostate cancer rates

**Variable**	**Prostate cancer**	**Person-years**	**Cancer rate^a^**
Age at measurement			
20–24	158	2 242 795	7
25–29	464	2 255 138	21
30–34	1054	2 064 155	51
35–39	2142	2 233 231	96
40–44	3798	3 471 729	109
45–49	5198	2 411 995	216
50–54	5539	1 881 942	294
55–59	5470	1 448 056	378
60–64	4432	1 009 951	439
65–69	3162	642 324	492
70–74	1897	349 388	543
Year of birth			
<1900	2200	457 438	481
1900–1909	7666	1 733 605	442
1910–1919	11 117	3 390 484	328
1920–1929	8617	4 723 760	182
1930–1939	3071	4 035 564	76
1940–1949	611	4 250 296	14
⩾1950	32	1 419 558	2
Attained age			
20–29	0	765 591	0
30–39	3	2 231 577	0
40–49	126	4 670 388	3
50–59	1813	4 752 559	38
60–69	9511	4 178 645	228
70–79	15 429	2 645 662	583
80–99	6432	766 283	839
BMI (kg m^−2^)			
<18.50	147	130 103	113
18.50–19.49	367	335 470	109
19.50–20.49	914	790 346	116
20.50–21.49	1778	1 474 133	121
21.50–22.49	2912	2 215 710	131
22.50–23.49	3766	2 630 649	143
23.50–24.99	6983	4 248 713	164
25.00–27.49	10 001	5 156 901	194
27.50–29.99	4523	2 107 971	215
30.00–32.49	1453	672 027	216
32.50–34.99	370	176 482	210
⩾35.00	100	72 201	139
Height (cm)			
<160	245	138 073	177
160–169	7091	3 153 938	225
170–179	19 247	10 958 081	176
180–189	6421	5 367 993	120
⩾190	310	392 620	79
			
Total	33 314	20 010 705	166

a Number of prostate cancer cases per 100 000 person-years.

). Among these, a total of 33 314 prostate cancer diagnoses were observed. The mean age at diagnosis was 73 years and the overall mean BMI was 24.9 kg m^−2^.

In men with BMI below 35.0 kg m^−2^, the risk of prostate cancer was increasing with increasing BMI (Table 2

**Table 2:** Relative risk (RR) of prostate cancer with 95% confidence intervals (CI) from Cox regression analysis; age at measurement and birth cohort were included in the model in addition to either one of the categorisations of body mass index (BMI) or height

**Variable**	**Cancer cases**	**RR**	**95% CI**	**Test for linear trend^a^**
BMI (kg m^−2^)				
<18.50	147	0.92	0.78–1.08	
18.50–24.99	16 720	1.00	Referent	
25.00–29.99	14 524	1.07	1.05–1.09	
⩾30.00	1923	1.09	1.04–1.15	
BMI (kg m^−2^)				
<18.50	147	0.92	0.78–1.08	
18.50–19.49	367	0.91	0.81–1.01	
19.50–20.49	914	0.95	0.88–1.02	
20.50–21.49	1778	0.94	0.89–1.00	
21.50–22.49	2912	0.97	0.93–1.02	
22.50–23.49	3766	1.00	Referent	
23.50–24.99	6983	1.04	1.00–1.09	
25.00–27.49	10 001	1.07	1.03–1.11	
27.50–29.99	4523	1.07	1.02–1.12	
30.00–32.49	1453	1.09	1.03–1.16	
32.50–34.99	370	1.15	1.03–1.28	
⩾35.00	100	0.93	0.76–1.13	*P*<0.001
Height (cm)				
<160	245	0.64	0.57–0.73	
160–169	7091	0.89	0.87–0.92	
170–179	19 247	1.00	Referent	
180–189	6421	1.06	1.03–1.09	
⩾190	310	1.11	0.99–1.24	*P*<0.001

a BMI and height, respectively, were included as continuous variables.

), from an RR of 0.92 in men with BMI below 18.5 kg m^−2^ to 1.15 in men with BMI 32.5–34.9 kg m^−2^ compared with men with BMI 22.5–23.4 kg m^−2^ (reference group). An analysis stratified by age at measurement revealed that the excess risk of prostate cancer in obese men was roughly of similar magnitude irrespective of age at measurement (Table 3

**Table 3:** Relative risk (RR) of prostate cancer from the Cox regression analysis with 95% confidence intervals (CI) by age at measurement and body mass index (BMI) or height, adjusted for birth cohort

	**Age at measurement (years)**											
	**20–29 (622 cases)**		**30–39 (3196 cases)**		**40–49 (8996 cases)**		**50–59 (11 009 cases)**		**60–69 (7594 cases)**		**70–74 (1897 cases)**	
**Variable**	**RR**	**95% CI**	**RR**	**95% CI**	**RR**	**95% CI**	**RR**	**95% CI**	**RR**	**95% CI**	**RR**	**95% CI**
BMI (kg m^−2^)												
<18.50	0.93	0.41–2.08	1.02	0.59–1.76	0.72	0.48–1.09	0.77	0.55–1.08	1.17	0.90–1.51	0.53	0.29–0.99
18.50–24.99	1.00	Referent	1.00	Referent	1.00	Referent	1.00	Referent	1.00	Referent	1.00	Referent
25.00–29.99	1.20	1.00–1.44	1.06	0.99–1.14	1.06	1.02–1.11	1.09	1.05–1.14	1.08	1.03–1.13	1.08	0.98–1.18
⩾30.00	1.22	0.69–2.17	1.19	0.99–1.44	1.05	0.95–1.16	1.05	0.97–1.14	1.14	1.04–1.24	1.04	0.87–1.23
Height (cm)												
<160	0.56	0.08–3.97	0.42	0.18–1.02	0.52	0.35–0.78	0.70	0.56–0.88	0.65	0.54–0.80	0.60	0.43–0.83
160–169	0.73	0.53–1.01	0.91	0.81–1.03	0.91	0.85–0.97	0.90	0.86–0.95	0.91	0.86–0.95	0.87	0.79–0.95
170–179	1.00	Referent	1.00	Referent	1.00	Referent	1.00	Referent	1.00	Referent	1.00	Referent
180–189	1.14	0.97–1.35	1.04	0.96–1.12	1.04	0.99–1.09	1.02	0.97–1.08	1.10	1.02–1.19	1.16	0.97–1.37
⩾190	0.62	0.34–1.13	1.12	0.89–1.42	1.18	1.00–1.40	1.08	0.85–1.38	1.00	0.64–1.58	0.00	0.00–∞

). However, an indication of higher obesity-associated risk was seen in those being youngest at measurement. Men measured at the age of 20–29 years were younger than 70 years at the end of follow-up. In the analyses stratified by attained age (Table 4

**Table 4:** Relative risk (RR) of prostate cancer from the Cox regression analysis with 95% confidence intervals (CI) by attained age and body mass index (BMI) or height, adjusted for birth cohort and age at measurement

	**Attained age (years)**							
	**50–59 (1813 cases)**		**60–69 (9511 cases)**		**70–79 (15 429 cases)**		**80–99 (6432 cases)**	
**Variable**	**RR**	**95% CI**	**RR**	**95% CI**	**RR**	**95% CI**	**RR**	**95% CI**
BMI (kg m^−2^)								
<18.50	0.82	0.41–1.65	0.89	0.65–1.22	0.80	0.62–1.02	1.05	0.76–1.47
18.50–24.99	1.00	Referent	1.00	Referent	1.00	Referent	1.00	Referent
25.00–29.99	1.13	1.02–1.25	1.04	1.00–1.09	1.07	1.04–1.11	1.07	1.02–1.13
⩾30.00	1.58	1.29–1.94	1.00	0.91–1.10	1.07	1.00–1.14	1.04	0.93–1.15
Height (cm)								
<160	0.45	0.17–1.19	0.45	0.32–0.64	0.70	0.59–0.82	0.63	0.50–0.79
160–169	0.83	0.72–0.97	0.88	0.83–0.93	0.92	0.89–0.96	0.84	0.80–0.89
170–179	1.00	Referent	1.00	Referent	1.00	Referent	1.00	Referent
180–189	1.03	0.92–1.14	1.09	1.04–1.15	1.07	1.02–1.11	1.02	0.95–1.11
⩾190	0.85	0.60–1.21	1.33	1.13–1.56	1.01	0.83–1.24	1.03	0.66–1.60

a Mean age at measurement (minimum–maximum) in the different strata: 50–59: 41 years (20–60); 60–69: 49 years (22–70); 70–79: 55 years (32–75); 80–99: 60 years (42–75).

), the RR of prostate cancer in age 50–59 years for obese men (measured at an average age of 45 years) was 1.58 (95% CI: 1.29–1.94) compared with men with BMI within the ‘normal range’.

An increasing risk of prostate cancer with increasing height was observed (Table 2). The tallest men had an RR of 1.72 (95% CI: 1.46–2.04) compared with the shortest men. The mean height was increasing with increasing birth year from 170 cm for men born around 1890 to 180 cm in those born around 1975 (mean annual increase: 0.12 cm). However, including men born before 1930 gave almost identical results with regard to the impact of both BMI and height on the risk of prostate cancer.

In order to reduce the potential effect of persisting disease at the weight measurement, analyses were performed with exclusion of the first 5 years of follow-up. This exclusion did not change the results. Since PSA testing was introduced around 1990, leading to an increase in the registered incidence of prostate cancer in Norway, analyses were performed using 31 December 1989 as the final date for the end of follow-up. Similar results were obtained.

Use of age-specific quintiles of height, weight, and BMI instead of the categorisation used above showed monotonely increasing risk of prostate cancer by increasing quintile. Compared with the lowest quintile, the highest quintile of height, weight, and BMI had RRs of 1.20 (95% CI: 1.16–1.25), 1.22 (95% CI: 1.18–1.26), and 1.13 (95% CI: 1.09–1.17), respectively.

## DISCUSSION

In the present study, height and weight were measured in 950 000 men, and these men were successively followed for an average of 21 years with respect to incidence of prostate cancer. An increasing risk of prostate cancer was observed both with increasing BMI and height.

Our study subjects were recruited from population-based studies with high attendance, and the measurements were performed in a standardised way. Use of a unique identification number and population-based registries on deaths, emigrations, and cancer incidence ascertained an almost complete follow-up of the study subjects (951 466 men). Only seven men were lost to follow-up. By the end of follow-up, 58.3% of the men were alive without a diagnosis of prostate cancer (0.9% of these had emigrated from Norway), 38.2% were dead, and 3.5% had a diagnosis of prostate cancer.

The present study is, to our knowledge, the largest study issued to explore the relation between height/BMI and prostate cancer, allowing a more detailed categorisation than in previous studies. The largest study so far on the association between BMI and prostate cancer (Rodriguez *et al*, 2001) included 820 000 men. The data were from the American Cancer Prevention Study I and II (CPS-I and CPS-II), and included 5200 prostate cancer deaths. A Swedish study (Andersson *et al*, 1997) included 2400 incident cases of prostate cancer and 700 deaths from prostate cancer. The present study included 33 300 incident cases.

The large study size made it possible to perform a number of stratified analyses, with high statistical power, which has not been possible in other studies on this issue. However, the stratified analyses were performed without having any specific hypothesis in advance. Therefore, the results must be interpreted with caution. The findings may be chance findings.

A stratified analysis by attained age revealed that there was a marked increased risk of prostate cancer in obese men compared with normal weighted men at age 50–59 years, while the RR in older age groups were modest. Since the incidence of prostate cancer is much higher at older ages, this effect, if it is real, will be hidden in studies where much of the total observation time is above this age. This might explain some of the inconsistencies between previous studies. In a recent review (Nomura, 2001), two of 10 cohort studies revealed a positive association between BMI and prostate cancer. These two studies (Andersson *et al*, 1997; Veierød *et al*, 1997) included relatively young populations. In one study (Veierød *et al*, 1997), using a subcohort of the present study (<3% of the men, <2% of the person-years), 91% of the person-years and 42% of the cases were less than 60 years. In the other study (Andersson *et al*, 1997), the association between BMI and both incidence of and mortality from prostate cancer was studied in a cohort of 135 000 Swedish construction workers followed for an average of 18 years; 53% of the men were below age 60 years during the entire follow-up period and additional 19% were below 60 years in most of the follow-up period. The association between BMI and prostate cancer was stronger when looking at mortality than at incidence. The study by Rodriguez *et al* (2001), not being included in the review by Nomura, found a positive association between BMI and prostate cancer mortality in both study cohorts. In CPS-I and CPS-II, the men had median age of between 52 and 57 years, respectively. The average follow-up was 12 years. In another study (Thune and Lund, 1994), not included in the review by Nomura, an increased risk of prostate cancer by increasing BMI in a cohort using parts of the present study cohort (<5% of the men, <3% of the person-years) was found. Also in this cohort, the men were relatively young with a median age at prostate cancer diagnosis of 61 years.

The four largest cohort studies reviewed by Nomura (2001), which did not find an association between BMI and prostate cancer, seemed to include a larger proportion of person-years above the age of 60 years than in the studies that found positive associations. In a Dutch study (Schuurman *et al*, 2000), 55–69-year-old men were followed for an average of 6 years. The median age at diagnosis was 70 years in the study by Habel *et al* (2000). Giovannucci *et al* (1997) followed 40–75-year-old men for an average of 7 years. A Norwegian study (Nilsen and Vatten, 1999), using a subcohort of the present study (2% of the men, 1% of the person-years), included men with a mean age of 59 years at the start of follow-up.

The majority of prostate cancer cases occur in men above the age of 60 years. However, the relative increase in prostate cancer incidence is higher in men aged 50–59 years than in other age groups both in the US as well as in other countries (Hsing *et al*, 2000). This can be both due to differences in screening practices by age, and the increasing proportion of obese men, suggested by an increased risk due to obesity in this age group in the present study.

Use of the Cox proportional hazards regression models assumes that RRs do not vary by time. The present finding of a somewhat different impact of obesity on the risk of prostate cancer at age 50–59 years violates the proportionality assumption in the Cox model. Restricting the analysis to ages above 60 years gave similar results as the main analysis, due to the low number of prostate cancer cases under the age of 60 years.

Some of the strengths of the present study were that height and weight were measured in various ages over a wide age-span and the long follow-up. Hence, we could adjust for age at measurement in the analyses. However, men being 80–99 years during the follow-up period had an average age at measurement of 60 years and only 11% were less than 50 years old. A possible explanation for a decreasing RR by attained age is that certain levels of BMI may have different meaning in young age and older age. However, persons with a BMI above 30 generally have a high percentage of fat, irrespective of age.

Some studies have looked at mortality from prostate cancer instead of incidence (Andersson *et al*, 1997; Rodriguez *et al*, 2001). Decreased survival among obese men has been suggested as an explanation for the stronger association between BMI and prostate cancer mortality (Rodriguez *et al*, 2001). Previous studies of the impact of BMI on the incidence of prostate cancer have been inconsistent, while studies on prostate cancer mortality have more consistently shown increased risk associated with obesity (Rodriguez *et al*, 2001). In the present study, we studied cancer incidence with the inclusion of histologically verified prostate cancers only.

The results of the present study on the relation between BMI and risk of prostate cancer were consistent with the estimates given by Bergström *et al* (2001). Use of the same intervals of BMI (BMI<20.0, 20.0–24.9, 25.0–29.9, and ⩾30.0) as Bergström *et al*, gave RRs of 1.07 (95% CI: 1.04–1.09) for men with BMI 25–29 and 1.09 (95% CI: 1.04–1.14) for men with BMI above 30 kg m^−2^ compared with those having BMI 20–24 kg m^−2^.

Even though only two of the nine cohort studies included in the review by Nomura (2001) found a significant positive association between height and prostate cancer, seven of the eight studies with an RR for height had RRs above one. Four (Andersson *et al*, 1997; Veierød *et al*, 1997; Nilsen and Vatten, 1999; Habel *et al*, 2000) of the six largest studies (Andersson *et al*, 1997; Giovannucci *et al*, 1997; Veierød *et al*, 1997; Nilsen and Vatten, 1999; Habel *et al*, 2000; Schuurman *et al*, 2000) found a 10–20% higher risk of prostate cancer in the highest quintile or quartile compared with the lowest. One study (Giovannucci *et al*, 1997) found an RR of 1.4 in the highest category compared with the lowest, while one study did not find any association at all (Schuurman *et al*, 2000). More recently, a positive association has been observed in CPS-I, but not in CPS-II (Rodriguez *et al*, 2001). In the present study, a markedly increased risk was found in the highest compared with the lowest men. The low risk connected to the shortest men can partly be explained by low incidence in the shortest men in the two northernmost counties in Norway. These two counties have a relatively large proportion of Lapps, who are generally short and have a low incidence of prostate cancer (Baste *et al*, 1992; Harvei, 1999). Categorising height by quintiles instead of the first chosen categorisation gave an RR of 1.20 (95% CI: 1.16–1.25) when comparing the highest with the lowest quintile. Excluding the two northernmost counties gave a slightly lower RR (RR=1.17; 95% CI: 1.13–1.21). The biological mechanisms underlying a possible association between height and prostate cancer are uncertain (Gunnell *et al*, 2001). A possible explanation is that height acts as a marker for the levels of insulin-like growth factors (IGFs) (Gunnell *et al*, 2001). An increased risk of prostate cancer has been observed in men with elevated plasma insulin-like growth factor-I (IGF-I) (Chan *et al*, 1998; Stattin *et al*, 2000).

In summary, in this large Norwegian cohort both height and BMI were positively associated with the risk of prostate cancer. The overall magnitude of the increase in risk of prostate cancer by increasing BMI was modest. However, in the age group 50–59 years, obese men had 58% higher risk than normal weighted men. The larger effect in this age group might partially explain the previous inconsistent findings. Even though the overall increased prostate cancer risk by BMI is minor, the large incidence of prostate cancer in older men in combination with the increasing BMI in many Western populations will increase the future importance of BMI as a risk factor for prostate cancer.
